# The ISI distribution of the stochastic Hodgkin-Huxley neuron

**DOI:** 10.3389/fncom.2014.00111

**Published:** 2014-10-08

**Authors:** Peter F. Rowat, Priscilla E. Greenwood

**Affiliations:** ^1^Institute for Neural Computation, University of CaliforniaSan Diego, La Jolla, CA, USA; ^2^Mathematics Department, University of British ColumbiaVancouver, BC, Canada

**Keywords:** ISI distribution, Hodgkin-Huxley, stochastic dynamics, stochastic differential equation, ISI histogram, Gillespie algorithm, Kurtz approximation

## Abstract

The simulation of ion-channel noise has an important role in computational neuroscience. In recent years several approximate methods of carrying out this simulation have been published, based on stochastic differential equations, and all giving slightly different results. The obvious, and essential, question is: which method is the most accurate and which is most computationally efficient? Here we make a contribution to the answer. We compare interspike interval histograms from simulated data using four different approximate stochastic differential equation (SDE) models of the stochastic Hodgkin-Huxley neuron, as well as the exact Markov chain model simulated by the Gillespie algorithm. One of the recent SDE models is the same as the Kurtz approximation first published in 1978. All the models considered give similar ISI histograms over a wide range of deterministic and stochastic input. Three features of these histograms are an initial peak, followed by one or more bumps, and then an exponential tail. We explore how these features depend on deterministic input and on level of channel noise, and explain the results using the stochastic dynamics of the model. We conclude with a rough ranking of the four SDE models with respect to the similarity of their ISI histograms to the histogram of the exact Markov chain model.

## 1. Introduction

Channel noise is important because it contributes to spike-time variability (Sigworth, 1980; White et al., 2000) and has been shown to be essential for subthreshold oscillations in stellate cells (White et al., 1998; Dorval and White, 2005), and for response variability in sensory cells (Fisch et al., 2012). In addition it contributes importantly to intrinsic irregular firing in cortical interneurons (Englitz et al., 2008; Stiefel et al., 2013), while in certain small neurons a single channel opening can initiate a spike (Lynch and Barry, 1989).

In this paper we compare published SDE approximation methods that simulate the stochastic Hodgkin-Huxley (HH) neuron model, by comparing the inter-spike-interval (ISI) distributions produced when driven by a constant DC current I. Theoretical work on the ISI distributions of stochastic neuron models was carried out by Chow and White (1996); Gerstein and Mandelbrot (1964); Gutkin and Ermentrout (1998); Tuckwell (2005), and Wilbur and Rinzel (1983).

**Figure 1:**
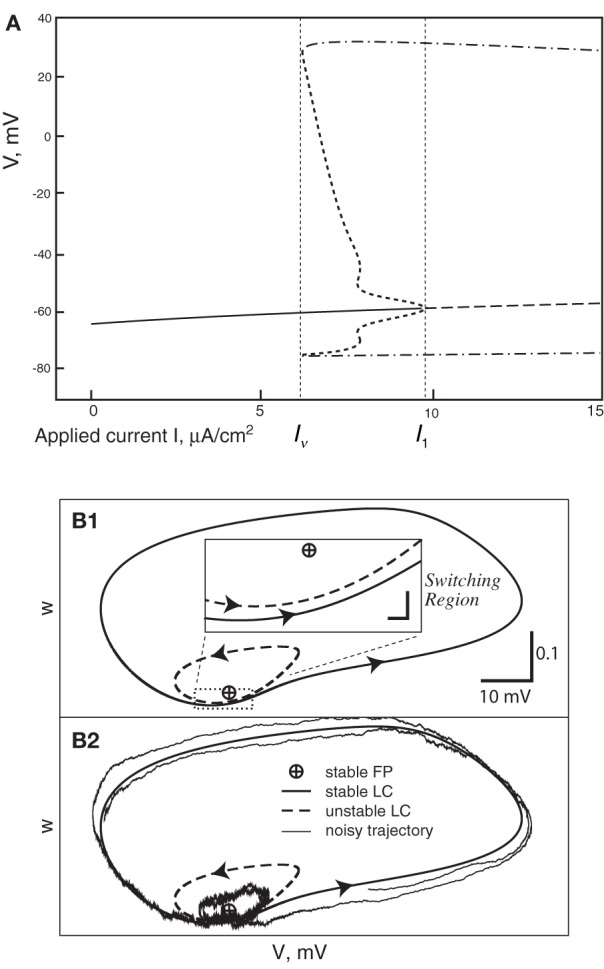
**(A)** Bifurcation diagram of the deterministic Hodgkin-Huxley model. Solid line, stable fixed point (SFP). Long-dashed line, unstable fixed point (UFP). Dot-dashed lines, extreme values of voltage on the stable limit cycle (SLC). Short-dashed lines, extreme voltage values on the unstable limit cycle (ULC). **(B)** The switching region in the two-dimensional Morris-Lecar model neuron. A similar, but four-dimensional, region is present in the Hodgin-Huxley phase-space. **(B1)** The fixed point is very close to the stable limit cycle with an unstable limit cycle (ULC) between them. Inset: the switching region, enlarged. **(B2)** This shows a noisy trajectory that emits one spike followed by sub-threshold oscillation inside the ULC and then another spike.

In all cases the deterministic model used as a basis for the various stochastic schemes is the classical model of Hodgkin and Huxley (1952). This model was introduced to describe action potentials in the squid giant axon, and remains a foundation of modern neuroscience. Its dynamics comprise a subcritical Hopf bifurcation together with a switching region in phase space where a fixed point is near to a limit cycle, the two being separated by an unstable limit cycle (Figures 1A,B). Thus, the deterministic HH model has a bistable range: when the input current, *I*, lies between 6.2 and 9.8 μA/cm^2^ (approximately) it is either spiking tonically—represented by the system traversing the locally stable limit cycle—or is quiescent—represented by the system spiraling inside the unstable limit cycle in toward the fixed point. When noise is present, and a trajectory traverses the switching region where the fixed point is close to the stable limit cycle, the system can switch between limit cycle behavior and quiescence. Thus, its overall behavior exhibits irregular switching between bursts of tonic spiking and periods of quiescence. This stochastic behavior continues to occur for a considerable range of the input, I, both below and above the deterministic bistable region *I* = [6.2, 9.8] (Yu and Lewis, 1989; Rowat, 2007).

Here we study the dependence of firing and quiescence patterns on the way noise is modeled, as reflected in the resulting distribution of inter-spike intervals (ISIs). The models we investigate are presented and studied in the papers by Fox and Lu (1994), Fox (1997), Goldwyn et al. (2011), Linaro et al. (2011), Orio and Soudry (2012), and Güler (2013).

In the standard stochastic model for the HH neuron each potassium channel has four binary gates, all of which must be open for potassium to be conducted. Each sodium channel has three activation gates and one inactivation gate, producing eight states, the channel being open only when it is in a particular one of these states [for complete details see Rowat (2007)]. The voltage dependent rates of moving between states have been established from data by Hodgkin and Huxley (1952).

A Markov chain algorithm for keeping track of the number of channels in each state was developed by Chow and White (1996), Gillespie (1977), and Skaugen and Walløe (1979). The algorithm was used by Rowat (2007) to compute several aspects of HH stochastic dynamics. This “exact” method of simulation of the stochastic HH we call the Micro model.

Because of both computation speed and ease of analysis, it is useful to replace the Micro Markov chain model with a system of stochastic differential equations, an SDE model. In fact we were shown how to do this already in a paper of Kurtz (1978), where a system of SDE's is constructed that approximates a density dependent Markov chain at a rate depending on population size N, with error of order log (*N*)/*N*.

Without knowing about the results of Kurtz (1978), authors of a number of papers, Fox (1997); Güler (2013); Linaro et al. (2011), and Orio and Soudry (2012), have devised systems of SDEs to approximate the Micro model. In fact Orio and Soudry (2012), using heuristics, derived the same set of approximating SDEs which a theorem of Kurtz (1978) defines for the Micro model. Here we call this model the Orio-Kurtz or simply Orio model, and sometimes the Kurtz approximation. However, it should be kept in mind that Kurtz proved the approximation for general Markov chain models in 1978. The complete Langevin system of SDEs proposed by Fox (1997), which requires taking two matrix square-roots at every time-step, was implemented for the first time by Goldwyn et al. (2011) so we sometimes refer to this as the Fox-Goldwyn model.

In neuroscience it is widely accepted that the distribution of spike timing, not simply mean spike frequency, is important. While the Micro, Fox-Goldwyn, Güler, Linaro, and Orio-Kurtz models all produce nearly the same mean spike frequency, it is not known how well these models capture the inter-spike-interval (ISI) distributions of the Micro model. Here we generate and compare the ISI distributions of the four SDE models with the ISI distributions of the Micro model, for a range of input current, I, that includes the region of bistability in the deterministic Hodgkin-Huxley model.

We find that, in fact, the ISI distribution is quite similar for all these models over a large range of (constant) deterministic inputs, I, and over a large range of channel numbers, which are proportional to A, the membrane area used. This result is, in fact, expected from analytic considerations, since, arguably, all the models are at least fairly well-approximated by the same diffusion process (Kurtz, 1978), which is based on a approximation theorem with a known error rate and can be regarded as a “gold standard.” We will see that both the Fox-Goldwyn and the Orio-Kurtz approximations give ISI distributions which are both quite close to that of the approximated Markov chain model. The Güler model compares well with the Fox-Goldwyn and Orio-Kurtz approximations while the Linaro model is somewhat less successful.

Our second main result is the first detailed description of the form of the ISI distribution of the stochastic HH, which appears in Section 3. In addition we explore how each of the features of the ISI distribution depends on I, the input to the model neuron, and on the number of channels in play, which is functionally related to the standard deviation of the noise in the system.

### 1.1. Four SDE models of Hodgkin-Huxley noise

The current conservation equation for voltage V (mV) and applied current *I* (μA/cm^2^) in the deterministic HH model is

(1) $$C\dot{V}=I-\left[g_{Na}(V-V_{Na})+g_{K}(V-V_{K})+g_{L}(V-V_{L})\right]$$

where the constants are given in Table 1.

**Table 1:** **Hodgkin-Huxley parameters for model simulations**.

C	Capacitance	1 μF/cm^2^
*g*_*K*_	Maximal potassium conductance	36 mS/cm^2^
*V*_*K*_	Potassium reversal potential	−12 mV
*g*_*Na*_	Maximal sodium conductance	120 mS/cm^2^
*V*_*Na*_	Sodium reversal potential	115 mV
*g*_*L*_	Leak conductance	0.3 mS/cm^2^
*V*_*L*_	Leak reversal potential	10.6 mS/cm^2^
ρ_*K*_	Potassium channel density	18/μm^2^
ρ_*Na*_	Sodium channel density	60/μm^2^
*N*_*K*_	Total number of potassium channels	ρ_*K*_ × Area
*N*_*Na*_	Total number of sodium channels	ρ_*Na*_ × Area

Equation (1) is the deterministic basis of the four stochastic differential equation (SDE) models we study: the Fox approximation, the Orio-Kurtz approximation, and the models of Güler (2013) and Linaro et al. (2011). Simulation of the Markov chain model called “ Micro” is detailed in Rowat (2007). A potassium channel has four activation *n*-gates, where each gate has the (opening, closing) rates (α_*n*_(*V*), β_*n*_(*V*)). The corresponding Markov network is

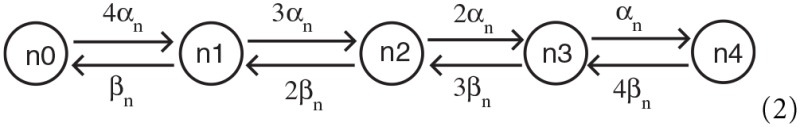



where a channel in state *n*_*i*_, *i* = 0, 1, …, 4, has *i* open *n*-gates. The channel is closed except when in state *n*_4_.

A sodium channel has 3 activation *m*-gates and one inactivation *h*-gate, where the *m*-gates have (opening, closing) rates (α_*m*_(*V*), β_*m*_(*V*)), and the *h*-gates have rates (α_*h*_(*V*), β_*h*_(*V*)). The corresponding Markov network is

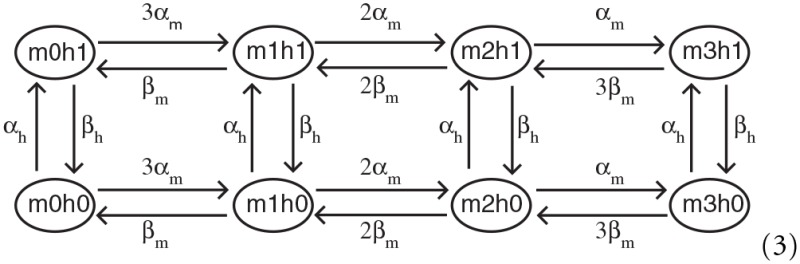



and the channel is open only in state m3h1. In state m*j*h*k*, there are *j* open *m*-gates and *k* open *h*-gates. The gate transition rates are given by the following functions:

(4) $$\begin{array}{ll} \alpha_{m}(V)=\frac{0.1(V+40)}{1-e^{-(V\;+\;40)/10}}, & \beta_{m}(V)=4e^{-(V\;+\;65)/18},\\ \alpha_{h}(V)=0.07e^{-(V\;+\;65)/20}, & \beta_{h}(V)=\frac{1}{1+e^{-(V\;+\;35)/10}},\\ \alpha_{n}(V)=\frac{0.01\left(V+55\right)}{1-e^{-(V\;+\;55)/10}}, & \beta_{n}(V)=0.125e^{-(V\;+\;65)/80}, \end{array}$$

In the Orio and Linaro models one writes an SDE for the proportion of channels in each of the states shown above. Because the K^+^ channels have 5 states and the Na^+^ channels have 8 states, these approximations consist of a system of 13 SDEs for Orio or 11 SDEs for Linaro, plus Equation (1), which has no explicit noise term.

In the K^+^-channel equations that follow, each variable *n*_*i*_ represents the proportion of channels in state *n*_*i*_, for *i* = 0, 1, …, 4. In the subsequent Na^+^-channel equations, for ease of notation we use variables *s*_0_, *s*_1_, …, *s*_7_ to stand for the proportions of channels in states m0h0, m0h1, …, m3h1. The correspondence is given in the Table 2.

**Table 2:** **Correspondence between si variables and states *m*jh*k***.

s_0_	s_1_	s_2_	s_3_	s_4_	s_5_	s_7_	s_7_
m0h0	m0h1	m1h0	m1h1	m2h0	m2h1	m3h0	m3h1

When Equation (1) is integrated, the values of the K^+^- and Na^+^-conductances are given by the definitions:

(5) $$g_{K}=n_{4}{\bar{g}}_{K},\;g_{Na}=s_{7}{\bar{g}}_{Na}$$

Here we write, in algorithmic form, how the SDEs which, in fact, follow from the Kurtz approximation (Kurtz, 1978), are formulated by Orio and Soudry (2012). A few words about the proof of the Kurtz approximation are in Section 1.2. To obtain each of the 13 equations for proportions of channels in each state, we first write the equation as an ordinary differential equation (ODE), thinking of the dynamics for a particular state as deterministic, i.e., the rates are deterministic input and outputs. For each state that is directly linked to the current state, we add to the right hand side a 2-component deterministic term with a positive input component and a negative output component. Then, for each of these deterministic terms on the right hand side, we add a noise term which has the form $\sqrt{x}$*dW* where *x*, in each case, is the deterministic term with any ‘−’ signs changed to ‘+,’ and *dW* is a Brownian increment. This gives the effective variance when the rates are considered as Poisson rates instead of deterministic rates. In each pair of directly linked states, the stochastic mass going out of one state is the same as the stochastic mass going into the other state. In these cases the terms $\sqrt{x}$*dW* are kept separate and have opposite signs in the two equations. We see examples in Equations (6) and (7) below. This simple description of the procedure for obtaining the Kurtz approximation (Kurtz, 1978) together with its justification is sketched in Greenwood and Gordillo (2009). Another version is in Orio and Soudry (2012), and its supplement S1.

The full system of SDEs for the potassium channel is given by:

(6) $$\begin{array}{l} {\dot{n}}_{0}=(-4\alpha_{n}n_{0}+\beta_{n}n_{1})+\frac{\xi_{1}}{\sqrt{N_{K}}}\sqrt{4\alpha_{n}n_{0}+\beta_{n}n_{1}}\\ {\dot{n}}_{1}=(4\alpha_{n}n_{0}-\beta_{n}n_{1})+(2\beta_{n}n_{2}-3\alpha_{n}n_{1})\\ \;-\frac{\xi_{1}}{\sqrt{N_{K}}}\sqrt{4\alpha_{n}n_{0}+\beta_{n}n_{1}}+\frac{\xi_{2}}{\sqrt{N_{K}}}\sqrt{2\beta_{n}n_{2}+3\alpha_{n}n_{1}}\\ {\dot{n}}_{2}=(3\alpha_{n}n_{1}-2\beta_{n}n_{2})+(3\beta_{n}n_{3}-2\alpha_{n}n_{2})\\ \;-\frac{\xi_{2}}{\sqrt{N_{K}}}\sqrt{3\alpha_{n}n_{1}+2\beta_{n}n_{2}}+\frac{\xi_{3}}{\sqrt{N_{K}}}\sqrt{3\beta_{n}n_{3}+2\alpha_{n}n_{2}}\\ {\dot{n}}_{3}=(2\alpha_{n}n_{2}-3\beta_{n}n_{3})+(4\beta_{n}n_{4}-\alpha_{n}n_{3})\\ \;-\frac{\xi_{3}}{\sqrt{N_{K}}}\sqrt{2\alpha_{n}n_{2}+3\beta_{n}n_{3}}+\frac{\xi_{4}}{\sqrt{N_{K}}}\sqrt{4\beta_{n}n_{4}+\alpha_{n}n_{3}}\\ {\dot{n}}_{4}=(\alpha_{n}n_{3}-4\beta_{n}n_{4})-\frac{\xi_{4}}{\sqrt{N_{K}}}\sqrt{\alpha_{n}n_{3}+4\beta_{n}n_{4}} \end{array}$$

Here ξ_*i*_, *i* = 1, …, 4 are Gaussian noise terms with mean 0 and standard deviation 1. Note that there are 5 SDEs but only 4 noise terms.

The SDEs for the sodium Markov network (3) can be read off directly from the network using the recipe described above between Equations (5) and (6):

(7) $$\begin{array}{l} {\dot{s}}_{0}=(\beta_{m}s_{2}-3\alpha_{m}s_{0})+(\beta_{h}s_{1}-\alpha_{h}s_{0})+\frac{\xi_{20}}{\sqrt{N_{Na}}}\sqrt{\beta_{m}s_{2}+3\alpha_{m}s_{0}}\\ \;+\;\frac{\xi_{10}}{\sqrt{N_{Na}}}\sqrt{\beta_{h}s_{1}+\alpha_{h}s_{0}}\\ {\dot{s}}_{1}=(\alpha_{h}s_{0}-\beta_{h}s_{1})+(\beta_{m}s_{3}-3\alpha_{m}s_{1})-\frac{\xi_{10}}{\sqrt{N_{Na}}}\sqrt{\beta_{h}s_{1}+\alpha_{h}s_{0}}\\ \;+\;\frac{\xi_{31}}{\sqrt{N_{Na}}}\sqrt{\beta_{m}s_{3}+3\alpha_{m}s_{1}}\\ {\dot{s}}_{2}=(3\alpha_{m}s_{0}-\beta_{m}s_{2})+(2\beta_{m}s_{4}-2\alpha_{m}s_{2})+(\beta_{h}s_{3}-\;\alpha_{h}s_{2})\\ \;-\frac{\xi_{20}}{\sqrt{N_{Na}}}\sqrt{3\alpha_{m}s_{0}+\beta_{m}s_{2}}+\frac{\xi_{42}}{\sqrt{N_{Na}}}\sqrt{2\beta_{m}s_{4}+2\alpha_{m}s_{2}}\\ \;+\;\frac{\xi_{23}}{\sqrt{N_{Na}}}\sqrt{\alpha_{h}s_{2}+\beta_{h}s_{3}}\\ {\dot{s}}_{3}=(3\alpha_{m}s_{1}-\beta_{m}s_{3})+(2\beta_{m}s_{5}-2\alpha_{m}s_{3})-(\alpha_{h}s_{2}-\beta_{h}s_{3})\\ \;-\;\frac{\xi_{31}}{\sqrt{N_{Na}}}\sqrt{3\alpha_{m}s_{1}+\beta_{m}s_{3}}+\frac{\xi_{53}}{\sqrt{N_{Na}}}\sqrt{2\beta_{m}s_{5}+2\alpha_{m}s_{3}}\\ \;-\;\frac{\xi_{23}}{\sqrt{N_{Na}}}\sqrt{\alpha_{h}s_{2}+\beta_{h}s_{3}}\\ {\dot{s}}_{4}=(2\alpha_{m}s_{2}-2\beta_{m}s_{4})+(3\beta_{m}s_{6}-\alpha_{m}s_{4})+(\beta_{h}s_{5}-\alpha_{h}s_{4})\\ \;-\;\frac{\xi_{42}}{\sqrt{N_{Na}}}\sqrt{2\alpha_{m}s_{2}+2\beta_{m}s_{4}}+\frac{\xi_{64}}{\sqrt{N_{Na}}}\sqrt{3\beta_{m}s_{6}+\alpha_{m}s_{4}}\\ \;+\;\frac{\xi_{54}}{\sqrt{N_{Na}}}\sqrt{\beta_{h}s_{5}+\alpha_{h}s_{4}}\\ {\dot{s}}_{5}=(2\alpha_{m}s_{3}-2\beta_{m}s_{5})+(3\beta_{m}s_{7}-\alpha_{m}s_{5})+(\alpha_{h}s_{4}-\beta_{h}s_{5})\\ \;-\;\frac{\xi_{35}}{\sqrt{N_{Na}}}\sqrt{2\alpha_{m}s_{3}+2\beta_{m}s_{5}}+\frac{\xi_{75}}{\sqrt{N_{Na}}}\sqrt{3\beta_{m}s_{7}+\alpha_{m}s_{5}}\\ \;-\;\frac{\xi_{54}}{\sqrt{N_{Na}}}\sqrt{\alpha_{h}s_{4}+\beta_{h}s_{5}}\\ {\dot{s}}_{6}=(\alpha_{m}s_{4}-3\beta_{m}s_{6})+(\beta_{h}s_{7}-\alpha_{h}s_{6})-\frac{\xi_{64}}{\sqrt{N_{Na}}}\sqrt{\alpha_{m}s_{4}+3\beta_{m}s_{6}}\\ \;+\;\frac{\xi_{76}}{\sqrt{N_{Na}}}\sqrt{\beta_{h}s_{7}+\alpha_{h}s_{6}}\\ {\dot{s}}_{7}=(\alpha_{m}s_{5}-3\beta_{m}s_{7})-(\alpha_{h}s_{6}-\beta_{h}s_{7})-\frac{\xi_{75}}{\sqrt{N_{Na}}}\sqrt{\alpha_{m}s_{5}+3\beta_{m}s_{7}}\\ \;-\;\frac{\xi_{76}}{\sqrt{N_{Na}}}\sqrt{\alpha_{h}s_{6}+\beta_{h}s_{7}} \end{array}$$

Güler (2013) presented a different stochastic Hodgkin-Huxley model. This model also approximates the stochastic dynamics of the membrane potential, arising from random opening and closing of sodium and potassium channels, by a system of seven differential equations, five of them stochastic, together with a modified version of Equation (1), appearing here as Equation (8). The stochastic dynamics, which follow, in a sense, more directly from the approach pioneered by Fox and Lu (1994) than the others, are approximated using carefully constructed diffusion coefficients. In addition, the drift coefficients contain stochastic components, *q*_*K*_ and *q*_*Na*_, designed to capture “non-trivial cross-correlation persistence” (NCCP) effects, namely correlations between transmembrane voltage fluctuations and the component of open channel fluctuations due to gate multiplicities (Güler, 2011). Since properties of the NCCP effects are similar to those of a harmonic Brownian oscillator, the equations that describe *q*_*K*_ and *q*_*Na*_ are written as those of a Brownian oscillator. Güler argues that NCCP effects have a major influence on excitability, spontaneous firing, and spike coherence. Güler reports that his model captures very accurately the functional correspondence between input current and mean spike frequency as obtained from the Micro structure (Markov network) model, as well as the mean spike frequency obtained from the Linaro model.

In the Güler SDE model, the current conservation Equation (1) is modified to read:

(8a) $$\begin{array}{l} C\dot{V}=-{\bar{g}}_{K}\psi_{K}(V-V_{K})-{\bar{g}}_{Na}\psi_{Na}(V-V_{Na})\\ \;-\;g_{L}(V-V_{L})+I \end{array}$$

(8b) $$\text{where }\psi_{K}=n^{4}+\sqrt{\frac{n^{4}(1-n^{4})}{N_{K}}}q_{Na}$$

(8c) $$\text{and }\psi_{Na}=m^{3}h+\sqrt{\frac{m^{3}(1-m^{3})}{N_{Na}}}hq_{Na}$$

and the periodic stochastic variables *q*_*K*_ and *q*_*Na*_ satisfy two second-order linear SDEs written as four first-order SDEs:

(8d) $$\tau {\dot{q}}_{K}=p_{K}$$

(8e) $$\tau {\dot{p}}_{K}=-\gamma_{K}p_{K}-\omega_{K}^{2}\left[\alpha_{n}(1-n)+\beta_{n}n\right]q_{K}+\xi_{K}$$

(8f) $$\tau {\dot{q}}_{Na}=p_{Na}$$

(8g) $$\tau {\dot{p}}_{Na}=-\gamma_{Na}p_{Na}-\omega_{Na}^{2}\left[\alpha_{m}(1-m)+\beta_{m}m\right]q_{Na}+\xi_{Na}$$

The gating variables n, m, and h are given by three more SDEs as in Fox and Lu's (1994) paper:

(8h) $$\dot{n}=\alpha_{n}(1-n)-\beta_{n}n+\eta_{n}$$

(8i) $$\dot{m}=\alpha_{m}(1-m)-\beta_{m}m+\eta_{m}$$

(8j) $$\dot{h}=\alpha_{h}(1-h)-\beta_{h}h+\eta_{h}$$

The Gaussian noise terms have zero means, with variances given by

(9a) $$Var(\xi_{K})=\gamma_{K}T_{K}\left[\alpha_{n}(1-n)+\beta_{n}n\right]$$

(9b) $$Var(\xi_{Na})=\gamma_{Na}T_{Na}\left[\alpha_{m}(1-m)+\beta_{m}m\right]$$

(9c) $$Var(\eta_{n})=\frac{\alpha_{n}(1-n)+\beta_{n}n}{4N_{K}},$$

(9d) $$Var(\eta_{m})=\frac{\alpha_{m}(1-m)+\beta_{m}m}{3N_{Na}},$$

(9e) $$Var(\eta_{h})=\frac{\alpha_{h}(1-h)+\beta_{h}h}{N_{Na}}$$

where the values of the fixed parameters in the Equations (8e,g) and (9a,b) are:

$$\gamma_{K}=\gamma_{Na}=10,\omega_{K}^{2}=150,\omega_{Na}^{2}=200,T_{K}=400,T_{Na}=800.$$

The functions α_*x*_ and β_*x*_, *x* = *n, m, h*, were given earlier in Equation (4). There are similarities between the Kurtz approximation and the Güler model, e.g., the Güler Equations (9c–e) specify that the diffusion terms of the SDEs (8h,i,j) are similar to the drift terms with ‘−’ changed to ‘+’ just as in the Kurtz approximation. There are significant differences seen in Güler's (8b,c) and in the fact that his SDEs (8d,e) form a second order SDE with a single noise term, and similarly for his SDEs (8f,g). Still it may be that Güler's model is an approximation to the Micro model in the same sense as the Kurtz approximation, or nearly so.

The Linaro model (Linaro et al., 2011) starts from the same current conservation Equation (1), appearing as Linaro et al. (2011; Equation 18). As in the Kurtz approximation, 11 SDEs are introduced Linaro Equation (19), but were obtained through the introduction of Orstein-Uhlenbeck processes for M-1 of the M elements of an M-state Markov process and applying this to the K- and Na- Markov processes. Hence both the drift terms and the diffusion coefficients take a different form from the Kurtz approximation Linaro Equation (19). In view of these differences it is perhaps surprising that the Linaro model produces ISI distributions which are rather close to those produced by the Micro model, the Fox-Goldwyn model, the Orio-Kurtz approximation, and the Güler model.

Diffusion approximations for this stochastic HH Markov chain model have also been studied by Bruce (2009); Goldwyn et al. (2011), and Huang et al. (2013). Engel et al. (2008) and Verechtchaguina et al. (2007) also study ISI histograms for a different modeled neuron and by a different approach.

### 1.2. Kurtz's strong approximation theorem for Markov chains

Here we describe briefly a theorem of Kurtz (1978) and how it applies to approximate the stochastic HH model by the system of SDE's consisting of Equations (1) and (4–7). A more complete version, including an alternate approach using a van Kampen expansion, is described by Baxendale and Greenwood (2011).

In fact one can approximate any normed density dependent Markov process, *X*^*N*^(*t*) = *X*(*t*)/*N*, with values in ℤ^*d*^, for large population size *N*, by a diffusion process with small error. The method of Kurtz (1978) represents a ℤ^*d*^-valued Markov process as a sum of Poisson processes. The essential step is replacing each normed compensated, or conditionally centered, Poisson process with a scalar Brownian motion, where an error of order log (*N*)/*N* is introduced. The resulting stochastic system can be written as

(10) $$d{\tilde{X}}^{N}(t)=F({\tilde{X}}^{N}(t))dt+\frac{1}{\sqrt{N}}C({\tilde{X}}^{N}(t))dW(t),$$

where *F* is the vector field of conditional means of the terms in Kurtz's sum, and the *d* × *d* diffusion coefficient matrix function *C*(*z*) is chosen so that *C*(*z*)*C*(*z*)^*^ = *B*(*z*), the covariance function arising from interactions of the terms. One avoids computing the square root of the matrix *B* by retaining the conditional centerings as separate Brownian increments as in Orio and Soudry (2012), Equation (13). We see these terms written out in Equations (5) and (6). This produces a sum of noise terms in each equation so that in distribution the system is the same as (9). The paper (Allen et al., 2008) gives the details of this process.

### 1.3. The form of the ISI distribution

Suppose we have a recording of membrane potential from a neuron firing in response to a fixed input current I, or we are looking at the output of a simulation of a neuron firing model such as one of those we are considering. An inter-spike interval (ISI) is the time between two successive downward crossings of the recording across a potential level chosen to be well above the range of sub-threshold oscillations. In general the successive ISIs of a simulation of a stochastic model are regarded as independent whereas those of a real neuron are not necessarily so. However, we do not pursue this question here. We are interested only in the distribution of the random ISIs.

The mean spiking frequencies of three models, Micro, Fox-Lu, and Güler are compared for a range of input currents in Figures 6–8 by Güler (2013). These means are not exactly the same but are rather similar. Here we look instead at the entire distribution of ISIs. In Figure 1A of Rowat (2007) we find, already, with Area = 100 μm^2^ and *I* = 0 μA/cm^2^, ISI histograms for the stochastic HH model considered by Chow and White (1996). Figure 16 of Rowat (2007) shows that the histogram of Figure 1A is nearly identical to that obtained when Gaussian noise is added to the HH current balance equation for a particular level of noise and a particular constant deterministic input. The effects of carefully modeled channel noise and an equivalent level of Gaussian noise added to the current balance Equation (1) are found to be nearly indistinguishable on the basis of the resulting ISI histograms. This observation motivates the present study where we compare ISI distributions more systematically and for additional recently studied SDE models of the stochastic HH equation.

**Figure 2:**
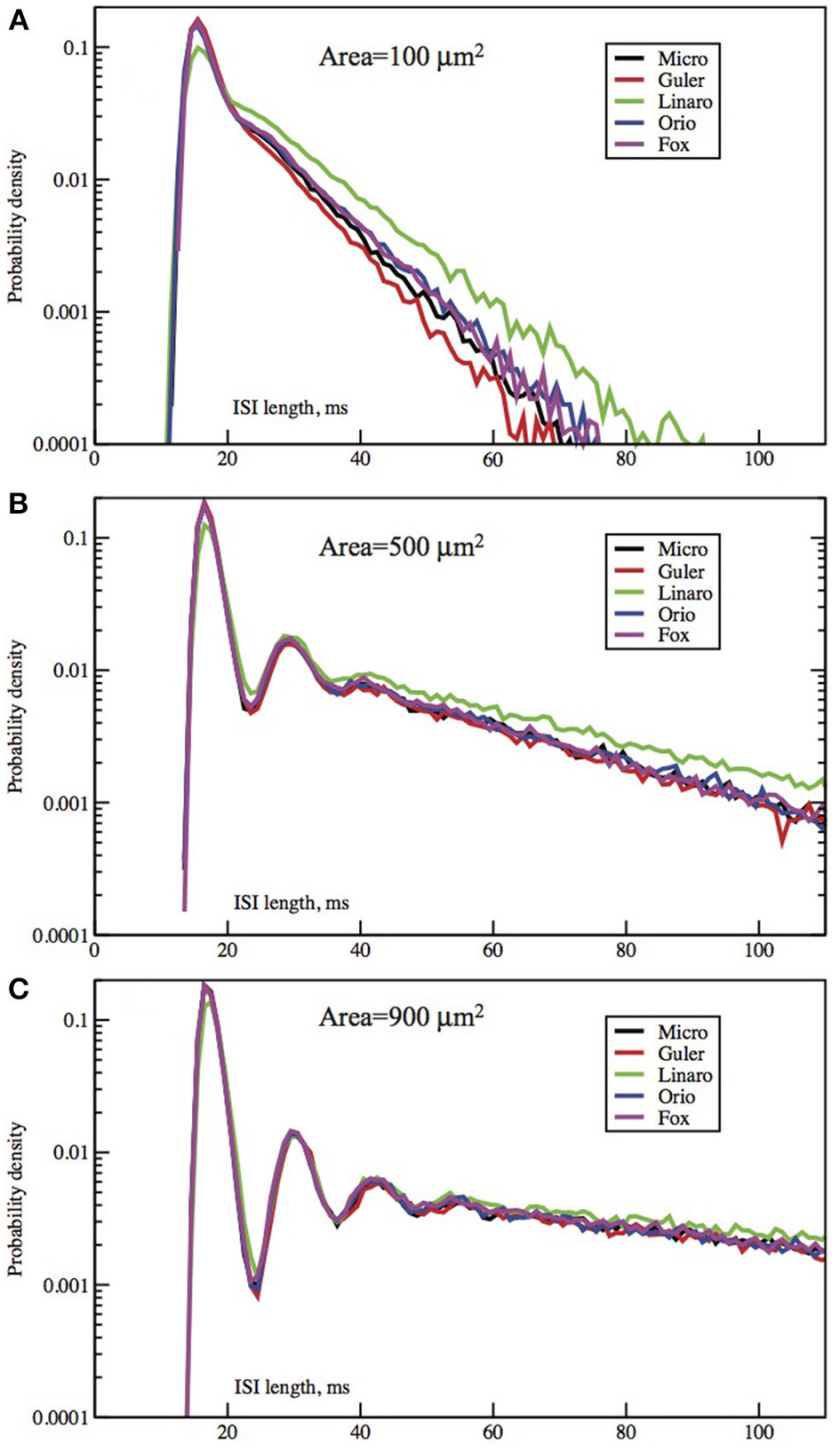
**Each panel compares the ISI distributions generated by the Micro, Güler, Linaro, Orio, and Fox methods, for a particular area, applied current combination**. Here, the current is constant at 6.0 μA/cm^2^ while the area takes values 100 μm^2^ **(A)**, 500 **(B)**, 900 **(C)**. Equivalently, the noise amplitude decreases from **(A to C)**.

**Figure 3:**
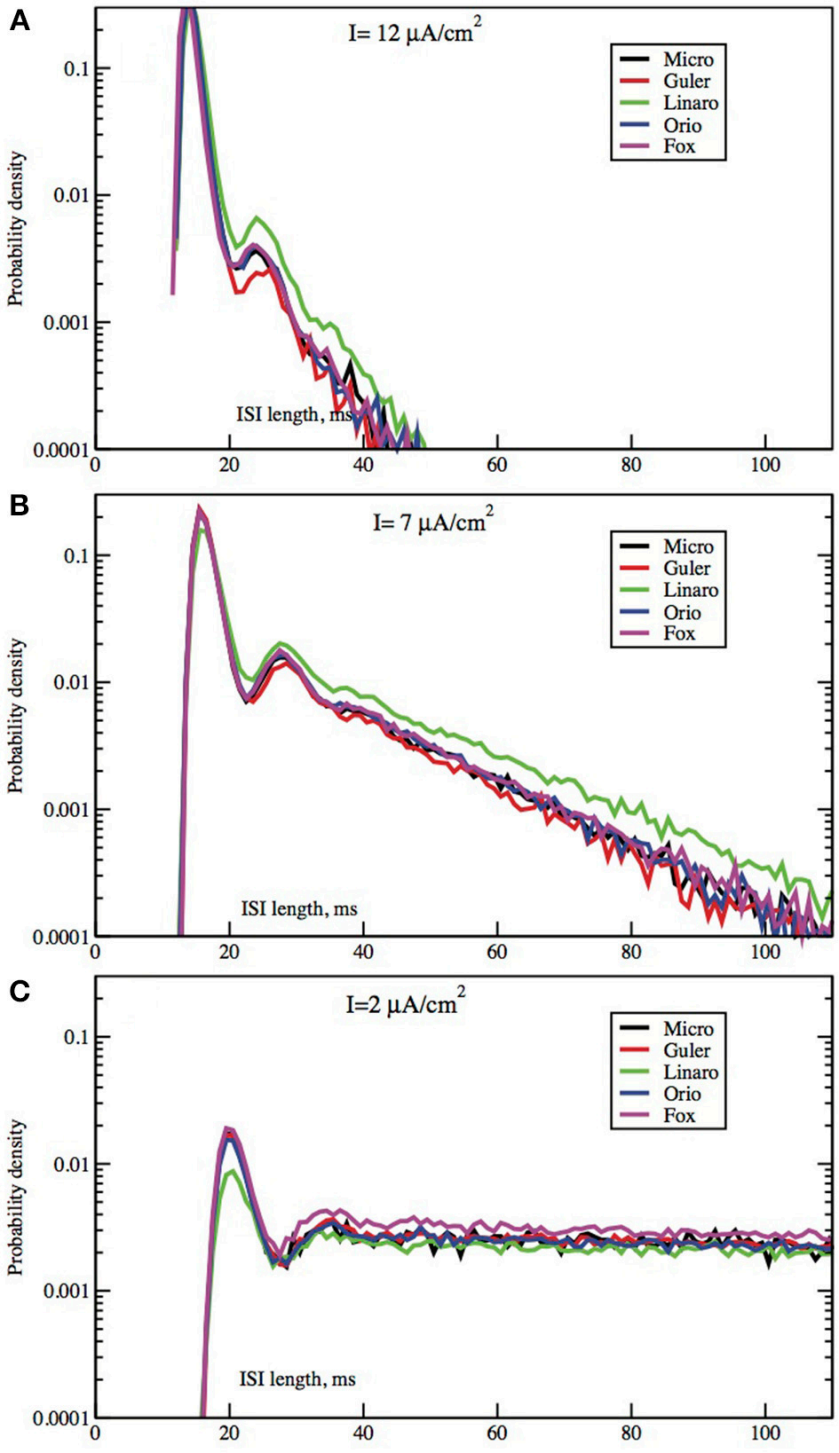
**Each panel compares the ISI distributions generated by the algorithmic methods Micro, Güler, Linaro, Orio, and Fox, for a particular area, applied current combination**. Here, the area is constant at 400 μm^2^ while the applied current takes values 10.0 μA/cm^2^ **(A)**, 6.0 μA/cm^2^ **(B)**, 2.0 μA/cm^2^ **(C)**.

The form of the ISI histograms indicates that the mean of the ISI distribution is in fact an inadequate parameter to use for comparison of stochastic models. The distribution is not unimodal but instead has the following characteristic form (see Figures 2, 3). For short time intervals there is a tall, narrow peak even on a log scale which represents the distribution of times taken by those individual spike firings which are preceded by one or more spikes, i.e., the times taken by the simulated stochastic path to traverse the locally stable limit cycle of the dynamics when there was a preceding spike. The fact that this first peak is narrow indicates that the variance of the time taken by a stochastic firing is small. The area under this first peak indicates the proportion of ISIs in runs, or “bursts,” of two or more spikes. As was found by Rowat (2007), Figure 4, the height of this spike increases with the deterministic input, I.

**Figure 4:**
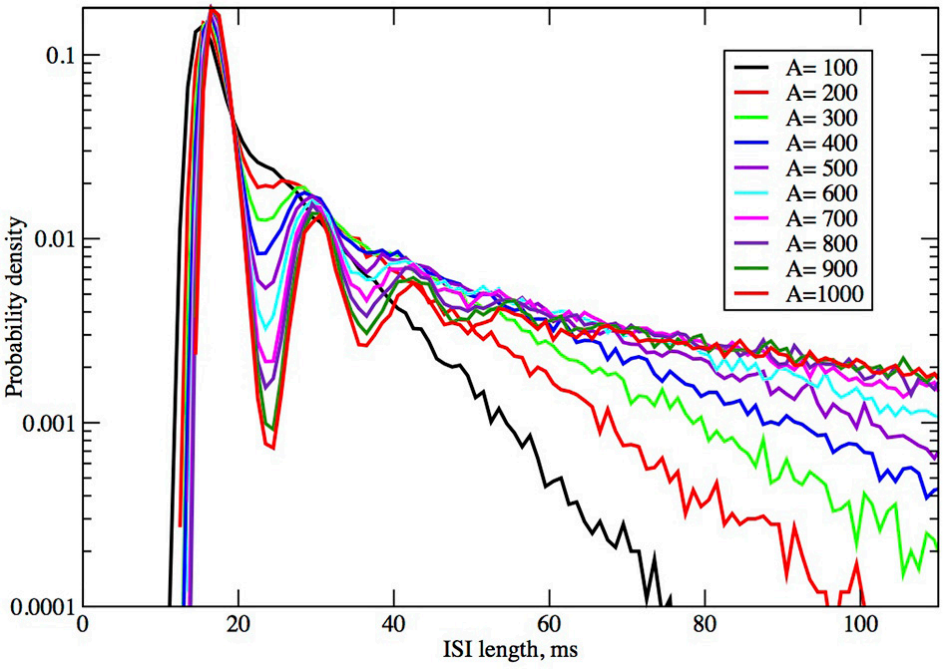
**ISI distributions obtained by the Orio-Kurtz method, for fixed current *I* = 6.0 μA/cm^2^, as area increases from 100 to 1000 μm^2^**.

The second obvious feature of the histogram, plotted on a log scale, as in Figure 16B of Rowat (2007) and Figures 2, 3, is that the right tail of the distribution is exponential, as indicated in our linear-log plots by a straight line, starting soon, though not immediately, after the initial peak. The histogram appears increasingly noisy as the length of the ISI time interval increases since the amount of data for estimation decreases, and because short histogram bars are enlarged by the log-scale.

The fact that the right tail of the ISI histogram is exponentially decreasing for the stochastic Morris Lecar model is studied in detail and explained by Rowat and Greenwood (2011). The explanation is based on the dynamics of the sub-threshold stochastic process which is in a conditional equilibrium beginning soon after a sub-threshold interval begins. The exit time from such an equilibrium must be exponentially distributed. This argument will apply to all stochastic HH models.

A third feature, less obvious but quite distinct, is that there are one or more small bumps or local maxima in the ISI histogram just after the initial peak, and before the exponential tail begins.

The one or more small local maxima in the ISI histogram are explained by considering the dynamics of the stochastic HH model just at the end of firing, i.e., as the stochastic path crosses into the basin of attraction of the locally stable fixed point of the deterministic HH model. The first orbit begins near the outer edge of the basin of attraction—the unstable limit cycle—and so the probability that the next firing (traverse of the locally stable limit cycle) comes after just one sub-threshold orbit is relatively high: see Figure 1B. Hence the probability that the ISI ends at the time taken by one such orbit from the end of the previous firing is relatively high. This produces the first small local maximum of the ISI distribution (see Figures 2, 3). Given that the next firing does not occur in this first small orbit of the fixed point, the next subthreshold orbit takes again a similar length of time, and again the probability of firing near the end of this second orbit is somewhat increased, producing the second local maximum, and so on. When there is less noise the pattern is more distinct. After one or two, or at most three such random orbits, the stochastic path is very nearly in its temporary, conditional stationary distribution concentrated close around the fixed point, so the remainder of the ISI distribution is exponentially distributed as discussed above. This description is made more explicit in the Morris Lecar two-dimensional system since the “inside” and “outside” of a limit cycle are well defined. In the four-dimensional HH system, the argument is made plausible by examining the relationships in 4 dimensions between the stable limit cycle, the unstable limit cycle, and the fixed point, and by projections onto a 2D plane (e.g., Rowat (2007), Figure 10, and the discussion).

The ISI distribution of any stochastic HH neuron model can, therefore, be resolved into three sections, an initial peak representing the distribution of times taken up by contiguous spike firings, followed by one or more local maxima representing the additional times taken up by each of a few subthreshold orbits of the fixed point immediately after firing, followed by an exponential tail representing time until escape from the sub-threshold state once the process is in its conditionally stationary distribution. Thus, a complete comparison of ISI histograms for simulations of stochastic HH models built from different noise models can be made by comparing the defining parameters of these three components: the center, height, and width of the initial peak, the shapes and placement of the local maxima, and the parameters of the exponential tails. We can use these criteria for comparing the ISI histograms produced by the four stochastic HH models described in Section 1.1.

## 2. Methods and implementation details

All model computer runs used the standard Hodgkin-Huxley parameter values, as in Table 1, and all data sets used for the ISI histograms had 10^5^ elements. All histograms were normalized so that their bars sum to 1, and all are displayed with a log scale on the y-axis because the first peak is often an order of magnitude higher than the second and third peaks. The integration time-step was 0.005 ms. An SDE was used for each state. If a potassium variable became negative the random number generator was called again. If a sodium variable became negative it was immediately reset to zero. At the end of each integration step, but before integrating Equation (1), the potassium variables *n*_*i*_, *i* = 0, …, 4 were normalized to satisfy ∑*n*_*i*_ = 1, and the sodium variables *s*_*j*_, *j* = 0, …, 7 were normalized by ∑*s*_*j*_ = 1. This seems more correct than defining the last variable (*n*_0_ or *s*_0_) in terms of the others, since it preserves the relative values of the variables, but has the disadvantage of using two extra SDEs. However, for the Orio method and any Kurtz-type approximation it does not increase the number of random number generator calls.

## 3. Results

**Figure 5:**
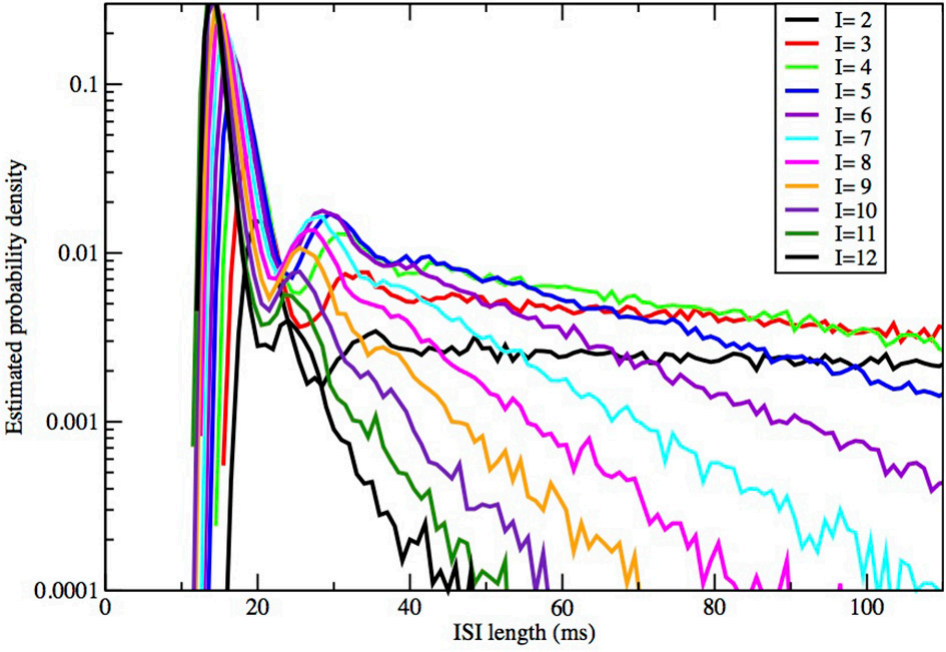
**ISI distributions obtained by the Orio-Kurtz method, for fixed area = 400 μm^2^ and current *I* = 2, 3, …, 12 μA/cm^2^**.

In Figures 2, 3 we see ISI histograms from simulations of the Markov chain model and the four SDE models, which are labeled Micro, Güler, Linaro, Orio, and Fox, to refer to the five ways of modeling HH channel noise described in Section 1.1. The noise level is proportional to Area^−1/2^ since the standard deviation of the Na^+^-channel noise (K^+^-channel noise) is ∝ *N*^−1/2^_*Na*_ (∝ *N*^−1/2^_*K*_) and the number of channels is a constant times the area. In Figure 5 where the area A = 400 μm^2^, *N*_*Na*_ = 60 × *A* = 24000 and *N*_*K*_ = 18 × *A* = 7200 Separate sets of plots show the results for fixed applied current *I* = 6 μA/cm^2^ and area *A* = 100, 200, …, 1000 μm^2^, and for fixed area A = 400 μm^2^ and applied currents *I* = 2, 3, …, 12 μA/cm^2^. See Figures 4, 5. All the histograms show the features detailed in Section 3: an initial peak, followed by local maxima, followed by an exponential tail, that appears linear on a log scale. The Güler, Micro, Orio, and Fox histograms are nearly identical in all respects and the Linaro plots are very similar.

The histograms computed by the Güler method all have a slightly higher proportion of area under the initial peak, corresponding to runs of successive spikes, than Micro-generated histograms, while Orio-generated histograms always have slightly lower proportion of ISIs in runs than Micro (differing by no more than 4%), while the Linaro histograms have proportions of ISIs in runs that are 8% lower than in Micro histograms.

Since the plots for the different SDE models are so similar it may not be worth dwelling on the differences. The exponential parameters of the tails are very close as evidenced by the nearly parallel plots. Also the timing of all features is nearly identical, showing that the different ways of modeling noise by SDEs have had little effect on the pattern of firing of the simulated neuron.

In Figures 2, 3 we see what happens to the three parts of the ISI histogram as the area A increases and the applied current *I* increases. Since A is proportional to the numbers of channels, in fact the standard deviation of the noise per unit time is proportional to A^−1/2^. We see in Figure 2 that the slope of the exponential part of the histogram, i.e., the slope of the last part, becomes less negative as A increases, equivalently, as the noise decreases. The height of the initial peak barely changes, but we see that the bumps in the middle part of the histogram become more prominent as noise decreases. When A is fixed and I, the deterministic input, decreases, in Figure 3 we see that the negative slope, i.e., the negative exponent of the exponential tail also decreases but, opposite to the case when noise decreases, the bumps (only one can be seen) become less prominent. The height of the initial peak decreases considerably, and its position moves right as *I* decreases. These effects are studied in more detail for the Orio model in Figures 4, 5. We discuss their interpretation in the next section, but give a summary in Table 3.

**Table 3:** **Changes in ISI distribution parameters with changes in noise level and applied current**.

**Parameter change**	**Height and position of main peak**	**Prominence of bumps**	**Negative tail exponent**
Increasing noise	Slight decrease in height; position moves left	Large reduction	Increases linearly
Increasing current	Large decrease in height; position has larger move left	Little change; bumps begin to disappear for *I* < 2	Increases super-linearly

## 4. Discussion

The models we have simulated produce similar histograms with the same basic features in good alignment. Here we discuss further how these features depend on two important parameters of the stochastic Hodgkin-Huxley model, the deterministic input, I, and the strength of the stochastic input which is proportional to A^−1/2^. Notice that these two parameters can be regarded as measures of deterministic and stochastic input, respectively.

**Figure 6:**
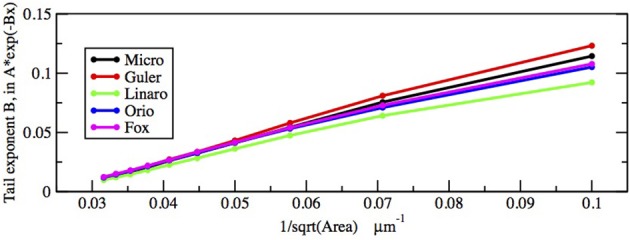
**Comparison of exponential tail exponents generated by the five methods, for fixed current *I* = 6.0 μA/cm^2^, plotted as a function of Area^−1/2^ where Area takes the values 1000, 900, …, 100 μm^2^**.

**Figure 7:**
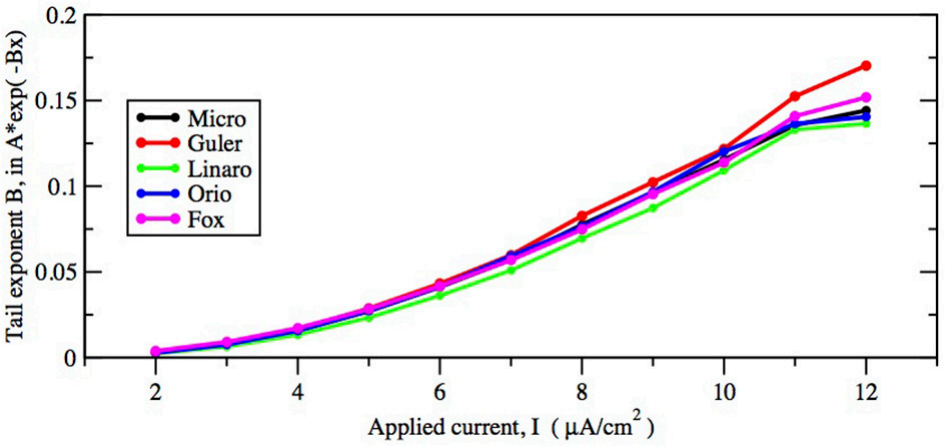
**Comparison of exponential tail exponents generated by the five methods, for fixed area *A* = 400 μm^2^, as current increases from 2 to 12 μA/cm^2^**.

First let us focus on how these two inputs affect the parameter of the ISI tail distribution. We see from the log-linear plots in Figures 2–5 that the negative slope of the final segment of the ISI distribution, which is the negative exponent of the exponential tail of the distribution, increases with increasing deterministic I, as well as with increasing stochastic input, A^−1/2^, i.e., these become steeper with decreasing A and with increasing I. In Figures 6, 7 the tail exponents are plotted as functions of A^−1/2^ and of I, respectively.

**Figure 8:**
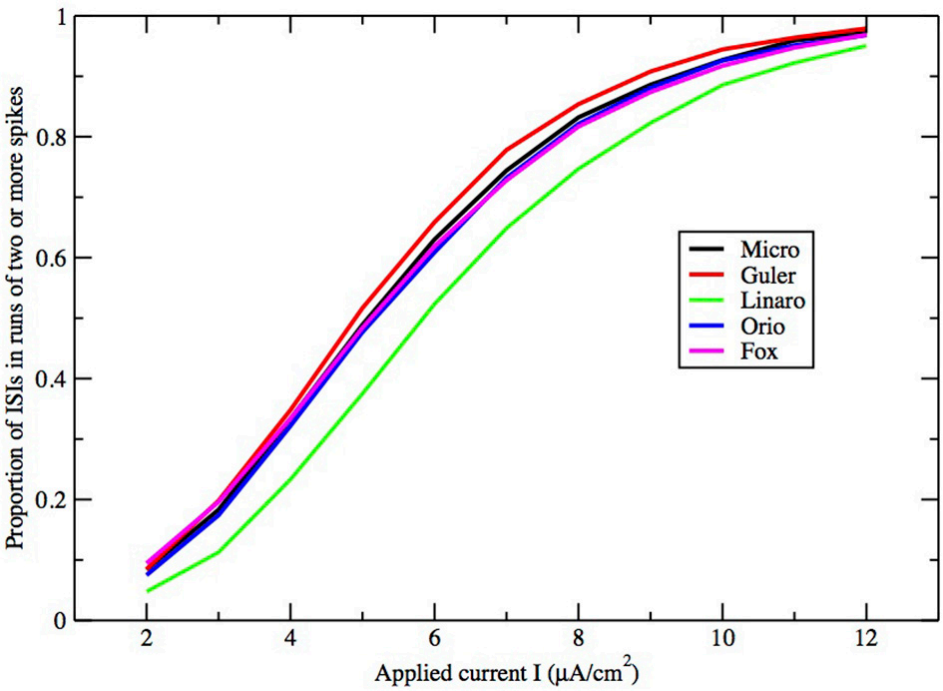
**Proportion of ISIs in runs of two or more spikes, compared by method, for fixed area *A* = 400 μm^2^, as a function of I**.

To understand this result we return to the state space picture of the stochastic dynamics of the neuron model, represented by Figure 1B2, which shows the dynamics for the analogous Morris-Lecar model. Between firings the state of the neuron is in the subthreshold region centered on the fixed point but well inside the unstable limit cycle except while traversing the switching region. The probability that it moves out of this region and fires is greater if either the noise has greater standard deviation or if the deterministic input to which the noise is added is greater. Hence an increase of either *I* or A^−1/2^ should have a similar effect on the parameter of the ISI exponential tail distribution. Furthermore, as *I* changes the configuration pictured in Figure 1B changes. As *I* moves toward the bifurcation at *I* ≈ 9.8, the bifurcation diagram in Figure 1A shows us that the stability of the fixed point decreases, becoming zero at *I* = 9.8, while the unstable limit cycle shrinks and disappears. Correspondingly, the subthreshold region shrinks in size, but does not disappear since one sees short intervals of subthreshold behavior for values of *I* ranging at least as high as 12.0. In Figure 8 one might note that when *I* = 12 none of the curves have reached 1. Both these effects cause the probability of firing to increase. Reduction in stability means it is easier to escape from the fixed point, while reduction in size of the unstable limit cycle means the size of the subthreshold regime is smaller, thus reducing the expected time to reach the deterministic basin of attraction of the stable limit cycle. The combination of these effects seem to cause the relation between *I* and the exponential tail to be concave, as in Figure 7, instead of nearly linear as in the case of noise, as in Figure 6. Switching to spiking and maintenance of spiking become more probable, and the exponential tail of the ISI distribution becomes steeper.

Next we consider the effect of increasing *I* or A^−1/2^ on the bumps in the middle part of the ISI histograms. To understand this we need to review some theory about the behavior of a similar neuron model during its subthreshold phase. This was studied in detail for the Morris-Lecar model by Ditlevsen and Greenwood (2013). The same analysis applies here with some alterations because the deterministic HH model is 4-dimensional. If we linearize the stochastic model at the fixed point we obtain a linear stochastic system of the form

(11) dX=−AX dt+C dW.

The deterministic matrix C is obtained by evaluating the stochastic diffusion coefficient matrix at the fixed point. The matrix A will have a pair of complex eigenvalues, −λ ± *i*ω with negative real part λ ≪ ω and other negative eigenvalues −β, −γ where β, γ are greater than λ. This means the system, started near the fixed point, moves rapidly toward the plane defined by the eigenvectors corresponding to the complex eigenvalues. Thus, the 4-dimensional system can be studied in terms of a 2-dimensional system. Other examples are found in Baxendale and Greenwood (2011).

When the neuron fires and then becomes subthreshold, the stochastic path enters the region “inside” the unstable limit cycle at its edge and proceeds to roughly circle the fixed point at a frequency ω, the orbit being damped at a rate λ while also being restrained from damping by the stochastic aspect of the model. The path of this process can be approximated in terms of a fixed rotation multiplied by a 2-dimensional Ornstein-Uhlenbeck process as shown by Baxendale and Greenwood (2011). The sample path of the process orbits the fixed point for some time, with frequency ω, until it arrives at its stationary distribution. Before stationarity sets in we see one or more decreasing “bumps” in the ISI histogram, with frequency ω, and after stationarity sets in we have the exponential tail, being the escape distribution from a stationary distribution by a standard argument.

As examples, according to computation by Hassard (1978): for *I* = 5, λ ± *i*ω = −0.097 ± 0.521*i*, −β = −0.129, −γ = −4.60; for *I* = 9, λ ± *i*ω = −0.015 ± 0.578*i*, −β = −0.137, −γ = −4.73. We find that the spacing between the second and third bumps in Figure 4, where *I* = 6, and also for larger areas (not shown), is approximately 12 ms, which is in rough agreement with 2π /ω ≈ 12.05 ms for *I* = 5 above. It is notable that the eigenvalue frequency ω, and thus the bump spacing, bears no particular relationship to the frequency of the unstable limit cycle (ULC), as computed by Rinzel and Miller (1980). Let *I*_1_ ≈ 9.8 be the subcritical Hopf bifurcation. For *I* close to *I*_1_, *I* < *I*_1_, the eigenvalue frequency and the ULC frequency are the same at approximately 90 Hz, but as *I* decreases toward *I*_*v*_ ≈ 6.26, ω decreases by only 9% while the ULC frequency decreases steeply from 55% from 90 Hz at *I*_1_ to approximately 40 Hz at *I* = 7.5 then smoothly reverses direction and increases back up to about 50 Hz at *I*_Υ_. One might also note the reduction in λ as *I* increases from 5 to 9, while β and γ are both much larger than λ, as predicted by Equation (10).

**Figure 9:**
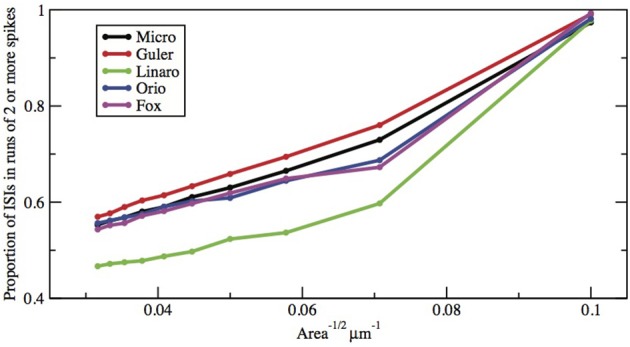
**Proportion of ISIs in runs of two or more spikes, compared by method, for fixed current *I* = 6.0 μA/cm^2^, as a function of Area^−1/2^**. Area takes values 1000, 900, …, 100 μm^2^.

Here we make a comment on the existence of exponential tails for *I* > *I*_1_. The underlying mechanism of this has been discussed in Rowat and Greenwood (2011). Numerically, it has been shown by Rowat (2007) and Tateno and Pakdaman (2004) that the probability *p*(*I*) that a spike is followed by a non-spike is continuous across *I*_1_. Note that 1 − *p*(*I*) is the proportion of ISIs in runs of two or more spikes (see Figures 8, 9).

When *I* > *I*_1_, say with *I* = 11 or *I* = 12, the equilibrium point is now unstable with dominant eigenvalues λ ± *i*ω, where λ is small, 0 < λ ≪ ω. Since λ is small one sees numerically that a deterministic trajectory started very close to the unstable equilibrium makes several very tight, small, slowly expanding spirals around the equilibrium before switching out to the stable limit cycle (SLC)—i.e., the spiking cycle.

When *I* is below the Hopf bifurcation, with dominant eigenvalues λ ± *i*ω where λ < 0 and −λ ≪ ω, Baxendale and Greenwood (2011) identify the stochastic process whereby deterministic damped oscillations, with the addition of noise, show sustained oscillations at an amplitude well above the expected noise level. The exit time from this stochastic equilibrium process is what creates the exponential tail when *I* < *I*_1_.

In view of the observation above, that when *I* > *I*_1_, several small tight spirals may occur before a deterministic trajectory, started close to the unstable FP, switches out to the SLC, it seems reasonable to propose that in the presence of noise there is a short-term stochastic process that tends to contract the slowly expanding deterministic spirals, thus creating a conditional equilibrium for a short time before the trajectory switches out to the SLC. Thus, the exit time from this conditional stochastic equilibrium has an exponential distribution that creates the exponential tail when *I* > *I*_1_.

The number and pronounced definition of the bumps become less as the noise increases because the onset of stationarity is hastened by more noise. We see this effect in Figure 4. In Figure 5 we observe that changing *I* with the noise level fixed has much less effect on the size and definition of the “bumps.”

Finally, how do the parameters *I* and *A*^−1/2^ affect the height, width, and location of the main peak of the ISI histogram? In Figure 8 we have plotted the proportion of ISI mass in runs of two or more spikes as a function of input *I* for fixed A = 400 μm^2^. This is represented in the histogram as the area under the first peak. It increases roughly linearly with *I* except for saturation at 0 and 1. The main peak moves right as *I* decreases and as A increases.

Note that any occurrence of a run of two or more spikes corresponds to the occurrence of a spike immediately followed by another spike, hence the proportion of ISI histogram mass under the first peak is in fact the probability that a spike is followed by another spike. Equivalently, the histogram mass or area under the tail (including any “bumps”) is the probability that a spike is followed by a period of quiescent behavior.

Figures 8, 9 show that the proportion of ISIs occurring in runs of two or more spikes increase roughly linearly with *I* and with *A*^−1/2^, respectively, when the other variable is fixed. This is reflected in the ISI distribution as an increase in the height and width of the initial peak. The reasons for increasing steepness of the exponential tail apply equally to the increase we observe in Figures 8, 9. A large negative tail exponent implies less area, or “probability mass,” under the tail and more mass in the main peak. Thus, the increases seen in Figures 8, 9 correlate well with the increases in negative tail exponent in Figures 6, 7.

In Table 4, we give numerical values for the exponential tail exponent, the proportion of ISIs in runs of two or more spikes, and the running times, across all the models, for one specific (Area, I) combination, namely Area = 400 μm^2^, *I* = 6.0 μA/cm^2^. These computer simulations were all run consecutively on the same hardware. We see that for these parameters, the Fox-Goldwyn and Orio-Kurtz methods are equally close (within a few percent), the Güler method a little further away, and the Linaro method further away again.

**Table 4:** **Parameters associated with each method, obtained from simulations with area = 400 μm^2^, *I* = 6.0 μ A/cm^2^, # ISIs = 10,000**.

**Method**	**Histogram tail exponent**	**Probability a spike is immediately followed by another spike**	**Compute time, 2.5 GHz Intel chip**	**Implementation**
Micro	0.04117	0.6302	84:34	C (gcc4.9)
Fox-Goldwyn	0.04164, 0.0005	0.6189, −0.011	28:28	Fortran95
Güler	0.04316, 0.0020	0.6587, +0.028	16:48	Python 2.7
Linaro	0.03614, 0.0050	0.5233, −0.107	52:10	Python 2.7
Orio-Kurtz	0.04133, 0.0002	0.6089, −0.021	45:44	Python 2.7

*Column 2 lists the exponential exponent of the histogram tail, while column 3 lists the probability that a spike is immediately followed by another spike. This is equivalent to the proportion of spikes that occur in a run of two or more spikes. In the lower four rows of columns 2 and 3, the first figure is the actual parameter value while the second figure is its difference from the corresponding value for the Micro simulation. The fourth column gives the running time on our hardware and the fifth the computer language used. Both the Fox-Goldwyn and Linaro code were retrieved from the ModelDB repository*.

ISI densities were also computed by Verechtchaguina et al. (2007) and Engel et al. (2008) by a different method and for a different neuron. An electrical circuit was used to capture the frequency-dependent subthreshold dynamics in stellate and pyramidal cells of the entorhinal cortex, which was converted to a noise-driven harmonic oscillator; from this they analytically computed ISI densities.

## 5. Conclusion

Figures 6–9 and Table 4 together show that the Fox-Goldwyn, and Orio-Kurtz methods both generate ISI histograms very close to those of Micro. The Güler histograms are not quite as close and the Linaro histograms are only a little further off.

According to Kurtz' theorem the Orio method gives an error of at most log (*N*)/*N* which is 0.0001 for the data sets computed here (*N* = 10^5^). Hence it should be regarded as a “gold standard” for producing a good approximation to the ISI distribution of the Markov chain model. However, when computation time is an issue, one might well prefer to use the Güler model which runs about three times as fast as the Linaro and Orio models. This was true for our Python implementations on a 2.5 GHz Intel Core i5 and will no doubt generalize to other languages and systems. We used the same basic code framework for the Güler, Linaro, and Orio methods. The main reason for the increased speed is that the Güler simulation calls the random number generator much less often than the others. In addition, the Güler method uses considerably fewer algebraic operations. Unfortunately the Fox-Goldwyn model was implemented in Fortran so its computation time cannot reasonably be compared with the other three SDE models.

Although the Güler method generates histogram parameters further away from the Micro histogram parameters than either the Fox-Goldwyn or Orio-Kurtz histogram parameters, one must bear in mind that when introducing harmonic Brownian oscillator-type SDEs, there are six phenomenological parameters in the Güler method that were carefully chosen by examination of simulations of Micro voltage data in a subthreshold regime, with *I* = −4 μA/cm^2^ (to avoid spikes). It may be that if these parameters were chosen with reference to Micro simulation voltage data generated with another *I*-value, e.g., in the middle of the bistability interval [6.2, 9.8], the parameters of the Güler histograms could be much closer to the parameters of the Micro histograms.

### Conflict of interest statement

The authors declare that the research was conducted in the absence of any commercial or financial relationships that could be construed as a potential conflict of interest.
